# Debate about evaluation and monitoring of sites carrying the HON-Logo

**DOI:** 10.2196/jmir.2.2.e13

**Published:** 2000-06-30

**Authors:** T Nater, C Boyer, G Eysenbach

## Letter

Sir,

The JMIR editorial article of March 31, 2000 [1], made a number of careless and erroneous remarks about the HONcode (http://www.hon.ch/HONcode/). HON welcomes criticism as long as it's constructive, verified, based on fact, and fair. Your comments meet none of these criteria.

We want to rebut three points in particular.

1. The editorial says, "even quackery sites proudly display the [HONcode] logo" and provides a screenshot of http://www.selfhealthsolutions.com/breakthrough.htm. Your assertion is seriously exaggerated and the "evidence" you use is out-of-date. We know about this site. Last year, we demanded an end to its fraudulent and unauthorized use of the HONcode seal. The site complied and has not displayed it since.

All systems are vulnerable to abuse, but HON has a good record of quickly identifying and contacting most offenders. This is thanks to the sense of shared responsibility we encourage among Webmasters, information providers, and vigilant users of the health Internet. The author, too, should have alerted HON to the misuse of the HONcode as soon as he discovered it, instead of saving this cheap shot for publication.

2. You wrote that "the HON-Logo is a 'marketing trick,' to make the HONcode well known." We resent the belittling tone of this remark and deny any such thing. HONcode remains the medical Internet's first and most widely-supported ethical standard. HONcode membership is entirely free and HON makes no income of any kind from it. The HONcode has been translated from English into 12 foreign languages and has more than 3,000 member Web sites in 36 countries - hardly the track record of a "marketing trick."

3. You wrote "[the HONcode] is a toothless tiger. A more sophisticated system is needed, for example where the logo or 'seal of approval' is generated dynamically by a third party (as planned in the medCERTAIN project described below)."

You are stretching the reader's credulity by comparing the HONcode, a tried and trusted four-year-old product, with your future "project." And you ignore the facts. JMIR readers should know that the HONcode hyperlink seal is indeed generated dynamically. We run a policing system with proven effectiveness and are in proactive, executive control of the process at all times.

HON reviews all sites during the formal membership application process, insists on necessary changes and improvements, and periodically monitors them for continuing compliance thereafter. We list reviewed HONcode-compliant sites on our MedHunt search engine. HONcode membership always implies attribution to the relevant site of a unique, randomly-generated six-figure ID number, for example, http://www.hon.ch/HONcode/Conduct.html?HONConduct166259for the National Library of Medicine's MEDLINEplus. The unique ID number, the Web site's URL, its HONcode registration status, correspondence and basic information on the site and its owner(s) are kept on a secure HON data base.

Finally, JMIR readers should be aware that we formally warn persistently non-compliant sites of the consequences of HONcode-related fraud and abuse. Our ultimate sanction is to de-link the HONcode seal on their pages. The vast majority of healthcare and medical Web sites prize their credibility and are striving to remain in business for the long term. This is why the threat of cut-off is usually enough to force problem sites either to comply with our demands or to remove our seal from their pages.

The HONcode will undergo a wide-ranging upgrade in the coming months to remain responsive to rapidly changing exigencies. While not a perfect system, it is the uncontested leader in its field. Too bad that the author of the JMIR article never sought to discuss with us some of the real challenges we face.

Timothy Nater, Executive Director

Celia Boyer, Manager of Web Services

Health On the Net Foundation, Geneva

### References

1. Eysenbach G: Towards ethical guidelines for e-health: JMIR Theme Issue on eHealth Ethics. J Med Internet Res 2000, 2:e7. [Full text]

### In reply

I regret that some of the remarks in the editorial [1] led to misunderstandings. As written in the editorial, the Health on the Net Foundation has been among the first to remind web publishers of their ethical duties, and is among the most successful initiatives in bringing these issues to a wide audience. Evidence-medicine has, however, taught us to stay critical; to continuously evaluate our interventions; and to ask how we can improve the effectiveness of our activities, especially if technology opens new possibilities. This letter gives me the opportunity to clarify some of the issues touched on in the editorial and to hopefully eliminate any misunderstandings.

Referring to point 1, "even quackery sites proudly display the [HONcode] logo": The figure showing a questionable website bearing a HON logo was meant to illustrate the inherent vulnerability and limitations of a system that relies on a "second generation" technology, i.e. a static logo which can be simply copied and pasted by webmasters (as opposed to third generation technology, i.e. a dynamically generated logo or a client-side tool interpreting metadata - see below). It is unquestionable that HON can deal with cases of abuse, once they come to their attention. The point I was trying to make was to suggest measures that minimize the risk of such abuse occurring in the first place, as opposed to a system that mainly relies on "post-marketing surveillance."

My proposal was that "the logo or seal of approval [could be] generated dynamically by a third party," suggesting a third generation of quality trustmarks, which can either be remotely loaded dynamic logos or PICS/XML/RDF-based metadata or both. In this approach, metadata and/or the logo would always come directly from the rating service to the user and could contain digital signatures, circumventing a necessary reliance on the co-operation of the health information provider (a number of sites carrying the HONCode-Logo have not actually included the dynamic backlink to HON).

**Table 1:** Definitions and Examples of Trustmarks

Generation	Characteristics	Examples
First generation trustmark	Logo/Award self-published by the information provider	HON up to 1999
Second generation trustmarks	Logo published by the information provider with hyperlink back to the rating service, generating a dynamic webpage	HON 1999, Etrust, Verisign
Third generation trustmarks	Metainformation coming directly from the rating service, either as PICS/XML/RDF metadata, or as dynamically generated seal	MedCERTAIN

The basic misunderstanding becomes apparent when the letter authors rebut this suggestion by saying that "you ignore the facts (...) the HONcode hyperlink seal is indeed generated dynamically." However, the HON seal (logo) itself is in fact *not* generated dynamically; only the page which will be generated if the user clicks the logo is dynamically generated. This is in fact a "second-generation" approach. A dynamic logo would be a logo which is for example remotely loaded from a third-part site, generated "on-the-fly," containing information such as a timestamp, the URL for which it is valid, and information on the status of the site. Or it could even be generated at the client-computer of the user, if a special software could interpret metadata retrieved automatically from the provider and the evaluator. The example in Figure 1 demonstrates such a dynamically generated logo with a timestamp, generated at the University of Bristol (a MedCERTAIN partner) in real time.

**Figure 1:** Example of a third-generation trustmark, a dynamically (on-the-fly) generated logo, with timestamp for demonstration (note that this is not the actual MedCERTAIN trustmark, but only an illustration). The logo is actually remotely generated and loaded from the University of Bristol. Websites evaluated by MedCERTAIN will include a code on their website which remotely loads the logo from the MedCERTAIN website, and the logo ("trustmark") can contain "real-time" information

Referring to point 2: Regarding the irritation of Nater & Boyer concerning my quote "As the Health on the Net Foundation says, the HON-Logo is a 'marketing trick,' to make the HONcode well known," two issues should be pointed out:

First, I should re-emphasize that the expression "marketing trick" was a quote (and indicated as such) from an individual involved in HON at a conference in 1998. The quote was used to remind readers that the original idea of letting information providers publish the HON-Logo was to promote the code, and not to allow users to check the status of the site. Only recently has HON asked information providers to include a hyperlink back to HON with an ID, providing the user with the possibility of verifying the status of the site.

Giving out logos or awards to other websites with a backlink to the originating site is indeed a very common marketing "trick" on the web, and frankly referred to as such (see for example http://www.saltocompany.nl/marktng.html). It is a legitimate marketing instrument to promote ideas, sites, products, or services. I doubt that the HONCode would have experienced a similar level of penetration if HON had relied on promoting the code in scholarly articles.

A second misunderstanding becomes apparent when the letter authors deny the idea of marketing and emphasize that HON "makes no income." The word "marketing" is commonly used in a nonprofit, public health context, without implying any financial purpose. Marketing has been defined (according to Kotler, quoted in [2], p. 29) as "human activity directed at satisfying needs and wants through exchange processes. An exchange process is simply the transfer, between two parties, of something that has value to each party. The marketer's task is to facilitate exchanges so that customers can fulfil their needs and wants. (...) Public sector and nonprofit marketers may benefit in nonmonetary ways, through the fulfilment of their institutional mission, desires, and goals." I see promoting the quality of health information on the Internet as a new challenge for public health in the information age, and "public health practitioners are in the business of marketing [2]." With these definitions in mind, I cannot see what can be considered pejorative about the word "marketing."

Referring to point 3: I did *not* write "[the HONcode] is a toothless tiger," as erroneously quoted in the letter. The actual wording was "without third-party evaluation and enforcement (...), this ethical code is a toothless tiger." " *This* ethical code" was in fact primarily referring to the Washington Code of eHealth ethics, which was the main topic of the editorial. Only three sentences of the editorial actually referred to HON. A clearer formulation would perhaps have been "without third-party evaluation and enforcement, *any* ethical code is a toothless tiger." It is noteworthy that a few weeks after publication of the editorial, a member of the Hi-Ethics group (another US group trying to implement a code of ethics for health websites) used almost the same words as used in the editorial:

Members of the group agree that devising enforcement mechanisms is the necessary next step. (...) "What doesn't work is a code with no follow-up and no teeth," [said Mark Boulding, a member of the group, in the context of the Hi-Ethics code] [3].

Neither this statement nor the statement in the editorial are meant to imply that either the HONcode or the Washington Code of eHealth ethics has no teeth, but only to point out that ethical codes have to be complemented by appropriate measures of evaluation and enforcement.

The question remains whether and to what extent sites carrying the HON logo are actually evaluated and monitored. The letter above suggests a rigorous evaluation, asserting that "most offenders" are "quickly identified." I would be interested in the actual percentage, i.e. how many "offenders" are identified per year, and what is the denominator? I.e. out of how many sites carrying the HON logo how many are not sticking to the HONcode? Shon & Musen [4] have recently shown that "HONoured" websites, found in HON's Medhunt, display authorship, currency of information, references, and disclosure of sponsors *less* often than a random selection of sites found in AltaVista. These data should be reason enough for HON to attempt to produce hard data on the actual quality of sites carrying the HON logo.

While the letter above suggest that sites registering at HON are evaluated in some way ("HON reviews all sites during the formal membership application process"), HON makes contradicting statements on their website (http://www.hon.ch/HONcode/), saying that "the HONcode is not an award system, nor does it intend to rate the quality of the information provided by a Web site. It only defines a set of rules to hold Web site developers to basic ethical standards in the presentation of information and to help make sure readers always know the source and the purpose of the data they are reading." If HON does not rate quality, on what grounds are quackery sites such as the one shown in the editorial's illustration asked to remove the HON-logo?

My main concern here is that, provided with remaining vague and sometimes contradictory information on this issue, the public can be easily confused. Even in the peer-reviewed literature, HON is sometimes referred to as a organization which rates the quality of websites, and a number of sites publish the HON-Logo under the heading "Awards" (which implies evaluation). MEDLINEplus, for example, which is cited in the letter, displays the HON-Logo under the heading "Awards." HON does not seem to regard this as "HON related fraud and abuse" and takes no measures to avoid this impression, although HON itself says that it is not an award system. This issue should not be taken too lightly. Consumers may erroneously mistake the HON-Logo as an award and rely on it as an indicator for assessed information.

The notion expressed by the authors in the letter - that issues concerning quality assessment were never discussed with them - is different from my own recollection. On the contrary, during my last conversation with one of the letter authors in Washington in February 2000, I had the impression that HON realizes the boundaries of their current approach and is willing to work with MedCERTAIN to improve the current system.

MedCERTAIN should not be seen as a competition or threat to HON, as it has a clearly different focus. While it also encourages self-regulation, it is very clear about the fact that it will actually rate (evaluate) websites and that it will build a technical platform for the interoperability of existing rating and reviewing services.

I made clear in the editorial and elsewhere [5] that both approaches (encouragement of self-regulation by HON, and third-party evaluation by organizations collaborating with MedCERTAIN) are important pillars towards quality management of health information on the Web, alongside educating users and enforcing "best practice" codes. We see the activities of MedCERTAIN as complementary to self-regulation efforts such as HON and the Washington eHealth Code of Ethics. We think that HON could well benefit from the technical and organizational infrastructure that will be provided by MedCERTAIN, and have from the beginning invited HON to join this collaboration. The future of monitoring, assessing, and evaluating health information certainly does not lie in reliance on a single, central organization, but in a democratic, decentralized, distributed and collaborative system [6].

Gunther Eysenbach

JMIR Editor-in-chief

**Disclosure:** Dr. Eysenbach is also co-ordinator of the EU project MedCERTAIN (Certification and Rating of Trustworthy and Assessed Health Information on the Net).
